# Enhanced lipid metabolism serves as a metabolic vulnerability to a polyunsaturated fatty acid (PUFA)-rich diet in glioblastoma

**DOI:** 10.21203/rs.3.rs-6355361/v1

**Published:** 2025-06-24

**Authors:** Prakash Chinnaiyan, Shiva Kant, Yi Zhao, Pravin Kesarwani, Kumari Alka, Jacob Oyeniyi, Ghulam Mohammad, Nadia Ashrafi, Stewart Graham, C. Ryan Miller

**Affiliations:** Corewell Health William Beaumont University Hospital; Corewell Health William Beaumont University Hospital; Corewell Health William Beaumont University Hospital; Corewell Health William Beaumont University Hospital; Corewell Health William Beaumont University Hospital; Corewell Health William Beaumont University Hospital; Corewell Health William Beaumont University Hospital; Corewell Health Research Institute; Corewell Health Research Institute; Heersink School of Medicine, University of Alabama at Birmingham

## Abstract

Enhanced lipid metabolism, which involves the active import, storage, and utilization of fatty acids from the tumor microenvironment, plays a contributory role in malignant glioma transformation; thereby, serving as an important gain of function. In this work, through studies initially designed to understand and reconcile possible mechanisms underlying the anti-tumor activity of a high-fat ketogenic diet, we discovered that this phenotype of enhanced lipid metabolism observed in glioblastoma may also serve as a metabolic vulnerability to diet modification. Specifically, exogenous polyunsaturated fatty acids (PUFA) demonstrate the unique ability of short-circuiting lipid homeostasis in glioblastoma cells. This leads to lipolysis-mediated lipid droplet breakdown, an accumulation of intracellular free fatty acids, and lipid peroxidation-mediated cytotoxicity, which was potentiated when combined with radiation therapy. Leveraging this data, we formulated a PUFA-rich modified diet that does not require carbohydrate restriction, which would likely improve long-term adherence when compared to a ketogenic diet. The modified PUFA-rich diet demonstrated both anti-tumor activity and potent synergy when combined with radiation therapy in mouse glioblastoma models. Collectively, this work offers both a mechanistic understanding and novel approach of targeting this metabolic phenotype in glioblastoma through diet modification and/or nutritional supplementation that may be readily translated into clinical application.

## INTRODUCTION

Glioblastoma (GBM) continues to be an invariably fatal malignancy with limited treatment options (1). Despite technological advancements and an unprecedented insight into molecular events contributing towards the growth of these tumors and its immune suppressive microenvironment, outcomes remain poor, with a median survival of less than 2 years and survival beyond 5 years uncommon (2). One key factor limiting the clinical advancements observed in other types of cancers to GBM is the blood brain barrier, which limits a majority of systemic therapies from attaining biologically relevant concentrations in the brain (3). Therefore, additional approaches that can build upon current treatment regimens that are effective, well-tolerated, and able to evade the blood brain barrier are needed to improve clinical gains in GBM.

Diet modification and/or nutritional supplementation has gained considerable attention from both the medical community and patients for its potential to prevent or treat a variety of illnesses, including cancer (4, 5), and represents a promising approach to enhance the effectiveness of standard therapies. The ketogenic diet (KD), which consists of a high-fat, low-carbohydrate diet, represents one of the most studied approaches. There have been numerous investigations demonstrating strong anti-tumor of the KD in GBM mouse models, both alone and in combination with radiation therapy (RT) (6–11). Mechanisms underlying this anti-tumor activity have not been definitively established, however, a majority of studies have attributed it to down-stream consequences of the low-carbohydrate aspect of this diet. Others have demonstrated the interface between diet modification and its impact on tumor growth to be more complex, including tumor cell energy homeostasis remaining unchanged following a KD(12) and the potential of the KD to have tumor promoting effects when extended to other models (13), suggesting further studies designed to better understand the interface between diet modification and tumor response are warranted. Clinically, there have been numerous retrospective studies(14–19) and early phase prospective studies(20–30) evaluating the KD in GBM patients and it is hopeful that ongoing, larger scale, Phase II studies will provide insight into its potential efficacy (31).

Our group and others have demonstrated that enhanced lipid metabolism plays an important role in GBM pathogenesis at multiple levels, serving as a ‘gain of function’ (32–41). For example, GBM cells have been shown to have the unique ability to actively import fatty acids (40), which are used to provide metabolic plasticity, allowing these cells to adapt to a dynamic microenvironement through fatty acid oxidation (41). However, seemingly paradoxical to these findings, as described above, significant antitumor activity has been observed in GBM when mice were fed a high fat/low carbohydrate KD. Therefore, we performed a series of investigations designed to provide insight into this apparent disconnect. Through these studies, we discovered that the high fat aspect of the KD plays a contributory role in its observed anti-tumor activity through dysregulation of lipid homeostasis in GBM cells, independent of carbohydrate restriction. We went on to show that this anti-tumor activity is primarily attributed to polyunsaturated fatty acids (PUFAs), offering a novel approach of targeting this metabolic phenotype in GBM through diet and/or nutritional supplementation that may be more readily translated into clinical application.

## RESULTS

### A ketogenic diet demonstrates anti-tumor activity and enhances RT response in GBM mouse models.

**Figure 1:**
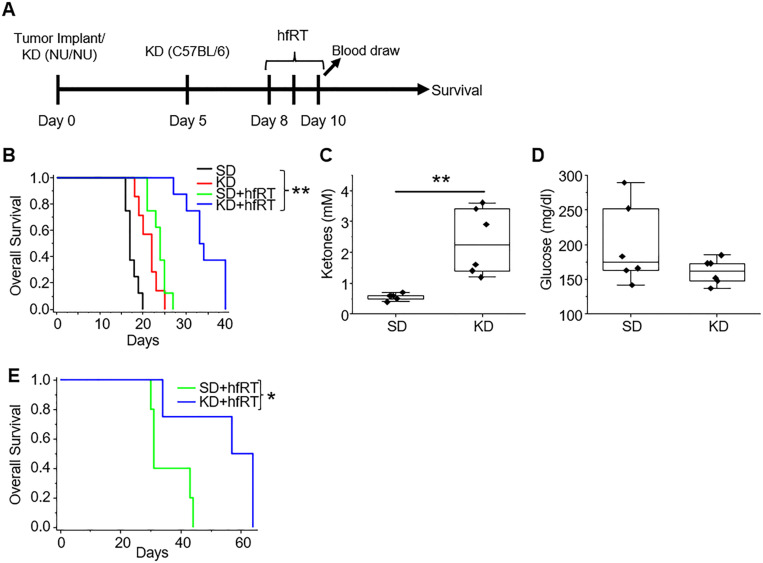
A ketogenic diet (KD) demonstrates independent anti-tumor activity and potent synergy when combined with hypofractionated radiation therapy (hfRT) in GBM. **A)**Treatment schema. **(B-D)** MES83 cells were orthotopically implanted in NU/NU mice (n=7–8 mice per arm) and **(E)** TRP cells were orthotopically implanted in C57BL/6 mice (n=4–5 mice per arm) and evaluated for survival and serum markers ketones or glucose following indicated treatment (n=6 mice per arm). Boxes represent the interquartile range, median and whiskers denote the upper and lower limits. Survival was determined by the Chi-square test. p values were determined by the log-rank test. *p<0.05; **p < 0.005.

As an initial investigation to begin to reconcile the reported, seemingly paradoxical findings of anti-tumor activity of a high fat KD in mouse GBM models in the context of a tumor where enhanced lipid metabolism serves as a gain of function, we evaluated the KD in a patient-derived, GBM tumor-initiating cell line MES83 that recapitulates the aggressive phenotype of this malignancy (41–43). In these studies, orthotopic mice model were either maintained on a standard diet or switched to a KD after tumor implantation, with or without radiation therapy (RT) (Fig. 1A). Irradiated mice were treated on Days 8–10 (6 Gy × 3 fractions) and blood was drawn to analyze ketones and glucose on Day 10. Consistent with previous reports, mice fed a KD demonstrated improved survival when compared to standard diet (median survival 17d vs. 22d; Fig. 1B). Interestingly, the growth delay offered by diet alone was similar to that observed when treated to a relatively high dose of RT in the context of preclinical models (median survival 23d). Although an improvement in survival was observed, tumors in these mice continued to grow, with all mice developing neurologic progression by day 27. Although individually, mice treated with RT or a KD alone resulted in a modest increase in survival, the combination demonstrated a dramatic improvement, resulting in a median survival of 33d with 40% of mice living past 35 days, reiterating the importance of developing combinatorial strategies in this aggressive, molecularly heterogeneous malignancy. As expected, ketosis was observed in mice fed a KD (Fig. 1C), however, similar to previous publications (24), a significant reduction in serum glucose levels was not observed after 10 days on a KD (Fig. 1D). A similar potential to enhance RT response was observed when we extended investigations to a syngeneic GBM mouse line TRP (44–47); Fig. 1E) and the ability of a KD to induce systemic ketosis without altering serum glucose concentrations in mice harboring this line has been previously published (8). Collectively, this supports the concept that mechanisms independent of the low carbohydrate aspect of the KD may contribute to the observed anti-tumor activity of a KD and its potential to enhance RT response.

### A ketogenic diet influences intra-tumoral metabolism in GBM.

**Figure 2:**
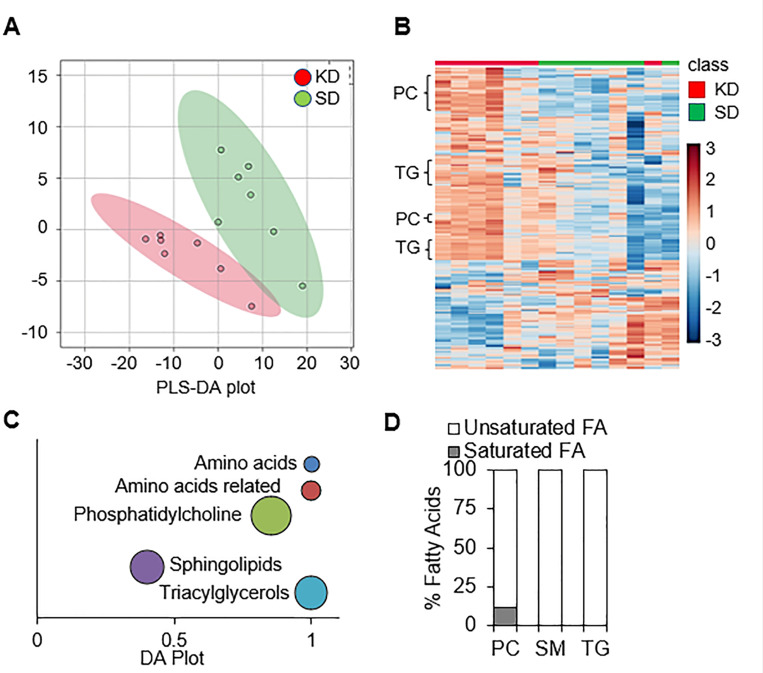
A ketogenic diet modulates intratumoral metabolism in GBM. Global metabolic proling was performed on MES83 tumors extracted from mice fed a standard diet (SD; n=7) or KD (n=7) and visualized by **(A)** PLS-DA score plot and **(B)** hierarchical clustering. **(C)** Significantly altered metabolites (p ≤ 0.05; Wilcoxon rank-sum test) in KD fed mice were classified into major metabolic categories and the differential abundance (DA) scores of each pathway plotted. Metabolic categories having more than three metabolites were used in the DA plot. Size of the bubbles are relative to the number of metabolites in each category. **(D)**Identified fatty acids for indicated metabolic categories (phosphatidylcholines [PC], sphingomyelins [SM], triacylglycerides [TG]) were classified as saturated or unsaturated fatty acids based on fatty acid chain.

As a KD leads to significant changes in systemic metabolism, we hypothesized these global changes would influence an individual tumor’s metabolism, potentially serving as a mechanism for its observed anti-tumor activity. To provide a window into the intra-tumoral metabolic consequences of a KD in GBM, we performed targeted metabolomic proling on GBM orthotopic tumors (MES83) from mice fed a standard diet or KD for 10 days. Using the commercially available biocrates MxP^®^ Quant 500 kit for multiplexed MS/MS analysis, a total of 255 metabolites were identified and quantified (Supplementary Table 1). Partial least squares-discriminant analysis (PLS-DA) indicated that the standard diet and KD groups were separated across two components (Fig. 2A), supporting the concept that diet modification can contribute to significant metabolic changes in an individual tumor. When analyzing the metabolomic data using unbiased hierarchal clustering by heatmap, clear separation was observed between metabolic pro les from mice fed a standard or KD (Fig. 2B). Interestingly, tumors from mice fed a KD demonstrated a significant accumulation of lipids, including phosphatidylcholines, sphingolipids, and triacylglycerides, which was recapitulated when plotted using differential abundance (DA) scores (Fig. 2C). As the accumulation of lipids emerged as a dominant metabolic node in GBM when mice were fed a KD, we went on to evaluate the identified fatty acids in further detail. Intriguingly, when looking at the characteristics of these fatty acids, we observed that an overwhelming majority of these lipids were unsaturated fatty acids (Fig. 2D).

### Polyunsaturated fatty acids modulate lipid dynamics in GBM.

**Figure 3:**
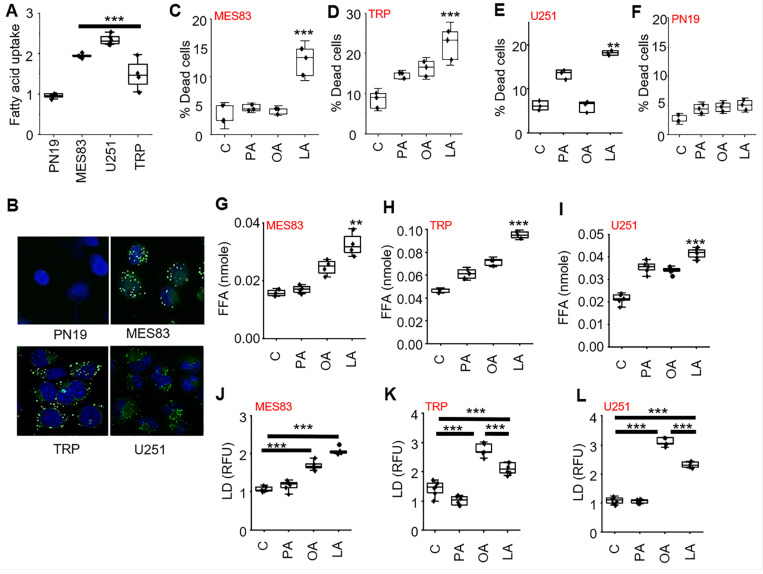
The polyunsaturated fatty acid linoleic acid modulates lipid homeostasis and induces cytotoxicity in GBM. **(A)** Fatty acid uptake was measured in real-time using c1-BODIPY-c12 in the indicated cell lines.**(B)** Cell lines were stained with BODIPY 493/503 and counterstained with DAPI to visualize intracellular lipid droplets using confocal microscopy. MES83 **(C,G,J)**, TRP **(D,H,K)**, U251 **(E,I,L)**, and PN19 **(F)** were treated with indicated fatty acids (palmitate [PA], oleic acid [OA], linoleic acid [LA]; 200μM). Non-viable cells were counted with trypan blue after 72 h. Free fatty acids (FFA) and lipid droplets (LD) were measured after 24h of the treatment. Boxes represent the interquartile range, median and whiskers denotes the upper and lower limit. Data is representative of three biologically independent experiments. **p < 0.005; ***p < 0.0005. RFU: Relative fluorescence unit. Data presented in Fig. 3D-I represent a portion of a larger experiment that was performed. This data is re-presented in Fig. 4B,C,E,F,H,I

We have previously described the unique capacity of GBM cells to actively import and utilize macronutrients, including lipids, from the microenvironment, a phenotype we termed enhanced metabolic heterotrophy (40). Therefore, we hypothesized this unique phenotype of GBM cells contributed to the observed intra-tumoral accumulation of fatty acids in mice fed a high-fat KD, and further, could potentially serve as a metabolic vulnerability. As an initial investigation, we sought to validate this phenotype in our GBM modes. Fatty acid uptake was measured in real-time (Fig. 3A) and visualized using immunofluorescent cytochemistry (Fig. 3B) with BODIPY labeling, demonstrating increased uptake in established GBM lines, including the aggressive mesenchymal line MES83, when compared to the proneural GBM line PN19, which we and others have previously shown to recapitulate the phenotype of low-grade glioma (41–43).

We next sought to determine if this uptake in fatty acids could contribute towards anti-tumor activity in these GBM lines, recapitulating the observed anti-tumor activity and lipid accumulation in tumors from KD fed mice. For these experiments, we tested a panel of fatty acids, including the saturated, mono-unsaturated (MUFA), and poly-unsaturated fatty acid (PUFA) palmitate, oleic acid, and linoleic acid, respectively. Interestingly, in all three GBM lines tested, only the PUFA linoleic acid led to a significant increase in cytotoxicity (Fig. 3C-E). Further, as expected, cytotoxicity was not observed in the proneural line PN19 (Fig. 3F), which does not recapitulate the metabolic phenotype of enhanced lipid metabolism/active lipid import observed in GBM. We went on to determine if the import of exogenous lipids influenced lipid homeostasis in GBM, postulating these exogenous fatty acids would lead to an accumulation of intracellular free fatty acids, which in turn, could contribute towards the observed cytotoxicity in GBM. Consistent with the anti-tumor activity observed in GBM lines, the PUFA linoleic acid was the only lipid that significantly increased the accumulation of free fatty acids (Fig. 3G-I). Next, we examined if these exogenous fatty acids impacted lipid droplet dynamics in GBM, which represent organelles that play an important role in lipid storage, regulation, and intracellular signaling. Although no change in lipid droplet formation was observed with the saturated fatty acid palmitate, both MUFA and PUFA (oleic and linoleic acid, respectively), led to a significant increase in lipid droplet formation (Fig. 3J-L).

**Figure 4:**
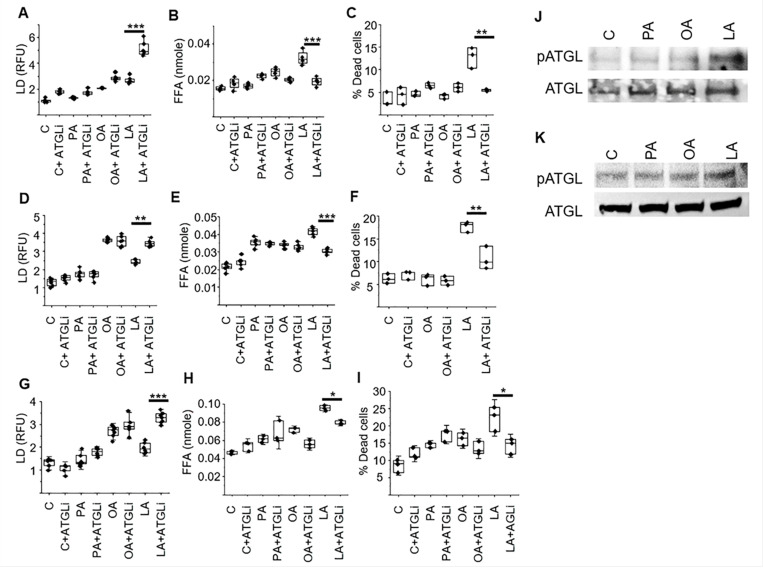
The polyunsaturated fatty acid linoleic acid modulates lipid droplet dynamics by activating lipase activity in GBM. MES83 (**A-C**) U251 (**D-F**) and TRP (**G-I**) were pretreated with +/− atglistatin (ATGLi; 25μM, 45 min) followed by treatment with indicated fatty acids (palmitate [PA], oleic acid [OA], linoleic acid [LA]; 200μM or control [C]). Lipid droplets (**A,D,G**; LD) and free fatty acids (**B,E,H**; FFA) were measured after 24h of the treatment. Non-viable cells (**C,F,I**) were counted with trypan blue after 72 h. Western blot evaluating indicated proteins and treatment conditions (8h) in U251(**J**) and TRP (**K**) cells. Boxes represent the interquartile range, median and whiskers denote the upper and lower limit. Data is representative of three biologically independent experiments. *p < 0.05; **p < 0.005; ***p < 0.0005. RFU: Relative fluorescence unit. The uncropped blots for 4J and 4K provided in Supplementary Materials.

Interestingly, in two of the three GBM models, there was less lipid droplet formation in the PUFA treated cells compared to the MUFA group (Fig. 3K-L), which was unique in causing both cytotoxicity and an accumulation of free fatty acids. We therefore explored how these different classes of fatty acids may differentially modulate lipid droplet dynamics in further detail. We postulated that this may be due to dysregulated storage of PUFAs in lipid droplets, resulting in an accumulation of free fatty acids and the observed cytotoxicity with linoleic acid in GBM. Lipolytic pathways play a central role in regulating lipid droplet dynamics and cellular lipid stores (48). Therefore, as an initial investigation, we sought to determine if exogenous fatty acids could modulate lipase activity in GBM, resulting in the observed changes in lipid droplet dynamics. To test this, we repeated the above-described studies, culturing GBM cells in a panel of fatty acids, with and without the non-specific lipase inhibitor diethylumbelliferyl phosphate (49); DEUP. This led to a further increase in lipid droplet formation, however, this was only observed in linoleic acid treated cells (Supplementary Fig. 1A-B), further supporting the notion that different classes of lipids differentially modulate lipid droplet dynamics in GBM. Studies were extended to determine if these observed changes in lipid droplet dynamics had biologic consequence. Consistent with our running hypothesis, DEUP normalized levels of free fatty acids (Fig. S1 C-D) and rescued GBM cells form the cytotoxicity of linoleic acid (Fig. S1 E-F), while having no impact on palmitate and oleic acid treated cells. As these studies used the non-specific lipase inhibitor DEUP, we went on to extend investigations to determine if the PUFA linoleic acid was directly targeting the lipid droplet by focusing on adipose triglyceride lipase (ATGL), which represents a central enzyme involved in lipid droplet degradation in mammalian cells (50), using the ATGL-specific inhibitor atglistatin (51). Consistent with the above findings, inhibiting the lipase ATGL was specific to the activity of the PUFA linoleic acid, leading to an accumulation of lipid droplets (Fig. 4A, D, G), a decrease in intracellular free fatty acids (Fig. 4B, E, H), and rescued cells from its anti-tumor effects (Fig. 4C, F, I) in all three GBM lines. Collectively, these findings support the concept that PUFAs are a unique class of fatty acids that demonstrate anti-tumor activity in GBM cells, which is mediated by differentially modulating lipid droplet degradation. We went on to evaluate how PUFAs modulate ATGL activity in further detail. Recent studies have demonstrated that phosphorylation of ATGL at Ser(404), which corresponds to murine Ser(406), leads to its activation and lipolysis (52, 53). We therefore went on to determine if ATGL phosphorylation was influenced by different classes of fatty acids. Indeed, in both human and mouse GBM lines, of the panel of fatty acids tested, only GBM cells treated with the PUFA linoleic acid demonstrated ATGL phosphorylation (Fig. 4J, K), further supporting the direct role PUFAs play in modulating lipid droplet dynamics in GBM.

## Modes of polyunsaturated fatty acid-induced cell death in GBM cells

**Figure 5:**
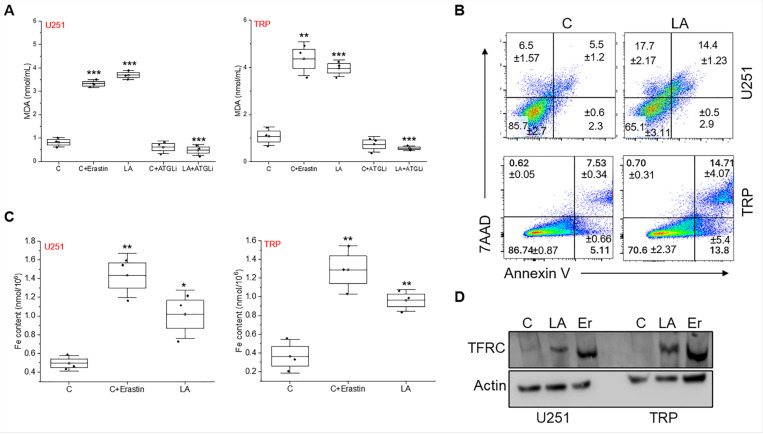
Modes of polyunsaturated fatty acid-induced cell death in GBM cells. (**A**) Indicated cell lines were treated with either vehicle control, erastin (10μM), linoleic acid (LA; 200μM) +/− atglistatin (ATGLi; 25μM) for 24h and evaluated for lipid peroxidation using the MDA assay. (**B**) Indicated cell lines were treated with either control or LA (200μM) for 72h and evaluated for apoptosis by Annexin V staining using flow cytometry. (**C**) Indicated cell lines were treated with either control, erastin (10μM), LA (200μM) for 24h and evaluated for ferroptosis by quantifying intracellular iron. (**D**) Western blot evaluating transferrin receptor (TFRC) expression in indicated cell lines treated with control, LA and erastin (Er). Data is representative of at least 2 biologically independent experiments. *p<0.05; **p<0.01; ***p < 0.001. The uncropped blots for 5D provided in Supplementary Materials.

We next explored how exogenous PUFAs contributed to the observed cytotoxicity in GBM cells. We demonstrated treating GBM cells with the PUFA linoleic acid led to an accumulation of free fatty acids, referred to as lipotoxicity (54). Interestingly, of the fatty acids, PUFAs are particularly susceptible to lipid peroxidation based on their unique carbon-carbon double bonds(55) and an increase in peroxidation can trigger multiple modes of cell death, including apoptosis(56) and ferroptosis, a form of iron-dependent cell death (57). We therefore evaluated these pathways in further detail. In both U251 and TRP lines, linoleic acid demonstrated an increase in lipid peroxidation, as measured by the MDA assay, at levels comparable to the positive control erastin, which represents an activator of lipid peroxidation and ferroptosis(58) (Fig. 5A). Consistent with the above studies demonstrating the ability of lipases to mitigate PUFA-induced accumulation of free fatty acids and cytotoxicity, atglistatin rescued GBM cells from PUFA-induced induction of lipid peroxidation. We went on to demonstrate multiple modes of cell death contributing to the anti-tumor activity of PUFA in GBM, including apoptosis (Fig. 5B) and ferroptosis (Fig. 5C-D), as measured by Annexin V labeling, iron content and transferrin receptor expression, respectively.

### Intra-tumoral heterogeneity of lipid metabolism in GBM

**Figure 6:**
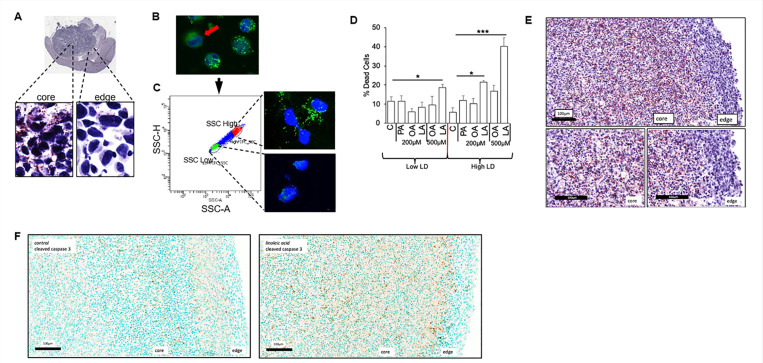
Intra-tumoral heterogeneity of lipid metabolism in GBM. (**A**) Oil Red O staining was performed on a MES83 tumor grown orthotopically in a mouse to evaluate for the regional accumulation of lipid droplets (LD). (**B,C**) Flow cytometry was used to sort MES83 LD^high^ and LD^low^ cells, based on side scattering which were then (**D**) treated with indicated fatty acids (palmitate [PA], oleic acid [OA], linoleic acid [LA] at stated concentrations). Non-viable cells were counted with trypan blue after 96 h. (**E**) MES83 cells were grown as organoids for 6 weeks and evaluated for LD using Oil Red O staining. (**F**) MES83 organoids grown for 6 weeks were treated with linoleic acid (200 uM) for 4 weeks, fixed, and immunohistochemical staining for cleaved caspase 3 was performed. *p < 0.05; ***p < 0.0005. Fig. 6B is derived from the same raw image used in Fig. 3B

We have demonstrated inter-tumoral heterogeneity of enhanced lipid metabolism in GBM lines, with this phenotype being present in all lines tested, except the proneural line, which despite being derived from GBM, harbors a metabolic phenotype more consistent with a low-grade glioma (40, 42). Interestingly, this molecular subtype has also been shown to be spatially enriched in the peripheral edge of an individual tumor (41, 59). We therefore explored the potential for intra-tumoral heterogeneity of enhanced lipid metabolism in GBM and sought to determine if this could influence the observed anti-tumor activity of PUFAs. Indeed, when evaluating for lipid droplets in GBM tumors grown orthotopically in mice, lipid droplets were observed more predominantly in the ‘core’ of the tumor when compared to the ‘edge’ (Fig. 6A), further supporting the intra-tumoral heterogeneity of this phenotype in GBM. To begin understanding the biological consequences of these findings, as an initial investigation, we evaluated for intra-tumoral heterogeneity in GBM cells grown in culture. Although a majority of cells in culture harbored the metabolic phenotype of enhanced lipid metabolism, which we de ne as the ability to import fatty acids and store in lipid droplets, we did identify the presence of small populations of cells in culture that did not (Fig. 6B). We hypothesized that even within this isogenic model, cells harboring this metabolic phenotype would be sensitive to the anti-tumor activity of PUFAs. To test this, we sorted GBM cells as lipid droplet ‘high’ and ‘low’ by ow-cytometry, which was confirmed by immunofluorescent cytochemistry (Fig. 6C). We allowed these sorted cells to grow in culture for 48h and, after confirming retention of their phenotype, treated them with the panel of fatty acids (Fig. 6D). Consistent with our hypothesis, anti-tumor activity was only observed in lipid droplet ‘high’ cells treated with the PUFA linoleic acid. Interestingly, although modest anti-tumor activity was observed in ‘low’ cells treated with a higher dose of linoleic acid (500 μM), a striking increase in cytotoxicity was observed in lipid droplet ‘high’ cells.

To allow us to further extend this line of investigation *in vitro*, we grew a GBM cell line as tumor organoids, which have been previously shown to more accurately recapitulate the tumor microenvironment, including developing regions of central necrosis, along with region-specific differences in lipid metabolism (37). When extending this approach to our model, we observed that GBM organoids recapitulated *in vivo* findings, with an accumulation of lipid droplets within the ‘core’ when compared to the ‘edge’ (Fig. 6E). We went on to test the hypothesis that ‘core’ cells harboring a phenotype of enhanced lipid metabolism would be particularly sensitive to the anti-tumor activity of the PUFA linoleic acid. Indeed, cleaved caspase activity was spatially enriched to the ‘core’ of organoids cultured with linoleic acid when compared to the ‘edge’ (Fig. 6F). Collectively, our findings demonstrate that there is clear intra-tumoral heterogeneity of enhanced lipid metabolism in GBM and that this phenotype may serve as metabolic vulnerability to PUFA-mediated cytotoxicity.

### Rationale combinatorial strategies to enhance PUFA-mediated cytotoxicity in GBM.

**Figure 7:**
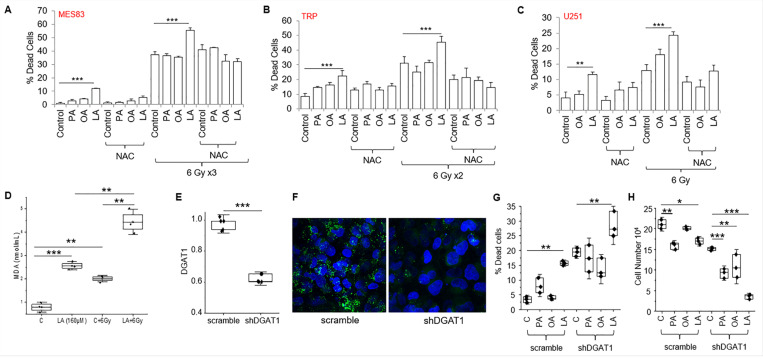
Combinatorial strategies to enhance PUFA-mediated cytotoxicity in GBM. **(A-C)** Indicated cell lines were treated with fatty acids (palmitate [PA], oleic acid [OA], linoleic acid [LA]; 200 uM) alone or in combination with N-acetylcysteine (NAC; 1mM) with and without radiation at stated dose. Non-viable cells were counted with trypan blue after 72–96 h. **(D)** U251 cells were treated with either vehicle control or linoleic acid (LA; 160μM) for 24h with and without RT (6 Gy) and evaluated for lipid peroxidation using the MDA assay. **(E)** Knockdown of DGAT1 expression in U251 cells using shRNA was validated using RT-PCR and **(F)** cells were evaluated for intracellular lipid droplets with BODIPY 493/503 using confocal microscopy. **(G-H)** Anti-tumor activity of the indicated fatty acids was evaluated in shRNA scramble control shDGAT1 U251 cells. Viable and non-viable cells were determined by trypan blue after 72 h. *p < 0.05; **p < 0.005; ***p < 0.0005. Data presented in Fig. 7B without NAC and/or RT is derived from data presented in Fig. 4I

Although we demonstrated clear anti-tumor activity of the PUFA linoleic acid in our panel of GBM cell lines, the observed activity was modest as a single agent. We therefore explored rational combinatorial strategies that may be utilized to potentiate this activity. As an initial investigation, we sought to recapitulate our in vivo data, which demonstrated strong synergy between the KD and RT. As demonstrated previously, anti-tumor activity was observed with the PUFA linoleic acid alone and as expected, irradiating MES83 GBM cells alone led to robust cytotoxicity (Fig. 7A). As hypothesized and consistent with our previous findings, of the panel of fatty acids tested, only the PUFA linoleic acid demonstrated the capacity to potentiate the anti-tumor activity of RT. Similar findings were observed when we extended this line of investigation to TRP and U251 cells (Fig. 7B-C). We have shown that PUFAs lead to lipotoxicity and lipid peroxidation in GBM cells, which was rescued with the lipid droplet lipase ATGLi. We hypothesized that oxidative stress plays a contributory role in both the observed anti-tumor activity mediated by the accumulation of these free fatty acids in GBM, and the potent synergy when combined with RT. To test this, we performed combination studies with the antioxidant N-acetylcysteine (NAC). In all three cell lines tested, NAC rescued cells from both the independent activity of the PUFA linoleic acid and when combined with RT (Fig. 7A-C).

As described above, PUFAs are particularly susceptible to free radical-induced lipid peroxidation based on their unique carbon-carbon double bonds. As the anti-tumor activity of RT primarily involves the generation of free radicals, we hypothesized this combination could contribute towards the observed synergy between RT and PUFAs. Using a slightly lower concentration of LA to more clearly demonstrate an interaction, the combination of LA + RT resulted in a significant increase in lipid peroxidation (Fig. 7D). Collectively, these findings support the concept that PUFA-mediated dysregulation of lipid metabolism results in anti-tumor activity in GBM cells, both alone and in combination with RT, through oxidative stress.

Next, as another strategy to enhance PUFA-mediated anti-tumor activity in GBM, we examined combinatorial approaches designed to modulate lipid droplet dynamics. Specifically, it has recently been shown that the storage of fatty acids into lipid droplets was regulated by the protein diacylglycerol-acyltransferase 1 (DGAT1) in GBM cells and its inhibition disrupted lipid homeostasis, resulting in high levels of reactive oxygen species and apoptosis (35). We therefore hypothesized that a cell’s inability to efficiently store fatty acids could render them more susceptible to the anti-tumor activity of exogenous PUFAs. Consistent with previous work, shRNA knockdown of DGAT1 in U251 cells inhibited lipid droplet formation (Fig. 7E/F) and demonstrated anti-tumor activity (Fig. 7G/H). The combination of shRNA knockdown of DGAT1 and only exogenous linoleic acid led to an additive increase in cytotoxicity (Fig. 7G). Interestingly, when evaluating for cellular proliferation, there appeared to be varying degrees of anti-proliferative effects in the fatty acids tested; however, the combination of shRNA knockdown of DGAT1 and exogenous linoleic acid led to a profound reduction in proliferation (Fig. 7H). This supports the concept that rationale combinations designed to target lipid droplet homeostasis may enhance therapeutic response in GBM.

### Evaluating the anti-tumor activity of a PUFA-rich diet in GBM *in vivo*.

**Figure 8:**
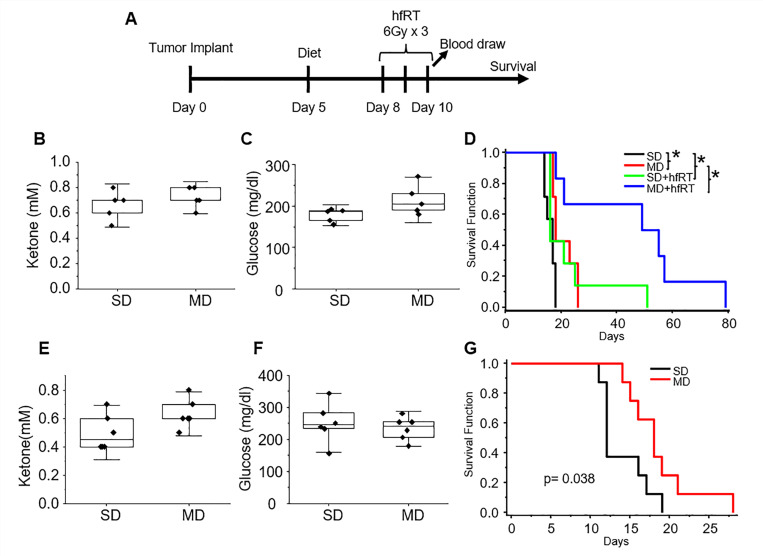
Evaluating the anti-tumor activity of a PUFA-rich diet in GBM in vivo. **(A)** Treatment schema. **(B-D)** U251 (n=6–7/group) and **(E-G)** TRP (n=8/group) tumors were grown intracranially in nu/nu and C57BL/6 mice, respectively, and treated according to the schema. Following the tumor implant, mice were fed either with followed by treatment with 6Gy of radiation on days 8,9 and 10 (hfRT) post-tumor implants as described in schema. Blood ketone and glucose concentrations were measured using the precision Xtra meter (B/C n=5/group; E/F n=6/group). Standard diet (SD), PUFA-rich modified diet (MD), hypofractionated radiation treatment (hfRT). *p < 0.05

In the work presented thus far, although both the anti-tumor activity of a KD and its capacity to potentiate RT response was confirmed in our GBM models, correlative studies coupled with systematic characterization of lipid droplet dynamics suggest that the high concentrations of fatty acids in this diet (namely PUFAs) play a contributory role in the observed anti-tumor activity. To extend this line of investigation *in vivo*, we designed a modified PUFA-rich diet (mPD) formulated to exploit the observed anti-tumor activity of these fatty acids in GBM and importantly, without requiring carbohydrate restriction, which will likely improve tolerability and clinical implementation of this approach. Specifically, the components of this more balanced diet consisted of 60%, 30%, and 10% kcal from fat, protein, and carbohydrates, compared to 90%, 9%, and 1% in a KD, respectively. Fat sources rich in PUFAs were used to prepare the modified diet, which constituted a significant portion of fat (1.92 kcal/gram), followed by monounsaturated (0.705 kcal/gram) and saturated fatty acids (0.341 kcal/gram). Further, the n6:n3 ratio in the mPD was within the recommended 1–3:1 to ratio (60). We therefore tested this PUFA-rich diet in our GBM models (Fig. 8A). The diet was well tolerated and there was no change in bodyweight in mice when compared to standard diet or a KD (Supplementary Fig. 2). As expected, there were no changes in systemic ketosis or glucose concentrations in serum from mice fed a mPD when compared to a standard diet (Fig. 8B/C). Consistent with our hypothesis, the mPD demonstrated both anti-tumor activity and potent enhancement of RT response in U251 tumors (Fig. 8D) and the anti-tumor activity of this diet was further validated in the TRP line (Fig. 8E-G). Collectively, this work supports the concept that the phenotype of enhanced lipid metabolism observed in GBM may serve as a metabolic vulnerability that may be therapeutically exploited by a PUFA-rich diet and/or supplementation.

## DISCUSSION

Diet modification has garnered considerable enthusiasm in the scientific community as a strategy to complement traditional cancer therapies to improve clinical outcomes (61). In addition, this approach has resonated with patients, as it provides them an opportunity to play a more active role in their cancer journey. This is particularly relevant in GBM, as scientific advancements have made limited gains in improving outcomes and diet modification could potentially circumvent the blood-brain barrier, which represents a considerable impediment to many, otherwise promising, therapeutics. Despite this level of enthusiasm, diet modification has yet to be deemed a ‘standard of care’ in the management of any type of cancer. Speci c to GBM, a majority of research involving diet modification has focused on the KD. Thus far, clinical testing has largely involved relatively small, pilot studies, however, an ongoing, larger scale Phase II study may finally provide insight into its potential efficacy in GBM (31). The wide-ranging adoption of diet modification as a bona de treatment strategy in cancer and its rigorous, prospective testing in the context of clinical trials, has been hindered by several factors. One factor is a limited understanding of underlying mechanisms contributing to the potential anti-tumor activity of a specific diet. This is further exacerbated by a lack of appreciation of the complex interface between systemic metabolism and the biology of an individual tumor, leading to an oversimplified perception of diet impacting a wide array of molecularly diverse types of cancers in a similar way. In GBM, several preclinical investigations have demonstrated potent anti-tumor activity of a KD (6–11), yet mechanistic underpinnings have yet to be clearly defined and are likely multifactorial. In terms of mechanism(s) of anti-tumor activity, a major emphasis has been on the low carbohydrate aspect of this diet, often dogmatically associating this with “starving” a highly glycolytic tumor. As our study and others have shown that serum glucose levels normalize quickly after initiating this diet, this perceived lack of glucose as a primary mechanism of anti-tumor activity is unlikely. However, other factors associated with carbohydrate restriction, including systemic ketosis and/or insulin growth factor signaling, may play a contributory role.

Our work, presented herein, was stimulated by metabolomic proling of GBM tumors from KD fed mice, which demonstrated significant metabolic changes within these tumors, most notable being an accumulation of PUFAs. Based on these results, we designed a series of experiments to determine if the high-fat aspect of a KD could independently play a contributory role in the observed preclinical activity of this diet. This line of investigation is supported by our previous work, which demonstrated the unique ability of GBM cells to actively import macronutrients from the microenvironment, including fatty acids and proteins, and utilize these substrates for survival and/or growth, a process we termed enhanced metabolic heterotrophy (40). Although this metabolic phenotype appears to serve as a gain of function in GBM cells, we postulated that it evolved in the context of a normal diet, and therefore, not requiring parallel molecular machinery to be in place to actively regulate this process. As it is well recognized that an overload of fatty acids can short-circuit intracellular lipid storage homeostasis, leading to disrupted cellular functions and lipotoxicity (54), we hypothesized that this unique phenotype observed in GBM cells may be exploited, serving as a metabolic vulnerability to a high-fat diet. Consistent with this hypothesis, exogenous fatty acids induced cytotoxicity in GBM cells harboring this metabolic phenotype. Interestingly, when tested using a panel of fatty acids, including the saturated, mono-unsaturated fatty acid (MUFA), and PUFA palmitate, oleic acid, and linoleic acid, respectively, anti-tumor activity in GBM cells, which resulted in an accumulation of free fatty acids and increase in lipid peroxidation, was specific to PUFAs. Our findings are consistent with a recent study by Dierge et al., which attributed these findings to increased lipid peroxidation induced by the acidic microenvironment in cancer (62).

Aberrant lipid metabolism, including the active import, storage, and utilization of fatty acids, has emerged as an important metabolic phenotype in GBM, contributing towards the aggressive phenotype this malignancy (32–41). As described above, the capacity to efficiently store these fatty acids in lipid droplets plays a critical role in maintaining this phenotype, as an accumulation of intracellular free acids can result in lipotoxicity. Accordingly, an accumulation of lipid droplets has been identified as a histologic hallmark of GBM (33, 35–37, 39). As our overarching hypothesis was that these exogenous fatty acids could disrupt lipid homeostasis in GBM, we studied these interactions in further detail. Interestingly, although both the MUFA and PUFA led to a significant increase in the formation of intracellular lipid droplets, only the PUFA linoleic acid, which was also the only fatty acid that demonstrated anti-tumor activity in GBM, resulted in lipotoxicity. Interestingly, treatment with the MUFA oleic acid led to a more significant increase in lipid droplet formation than the PUFA linoleic acid, prompting us to determine if these unique classes of fatty acids differentially modulated lipid droplet dynamics and/or homeostasis in GBM. As lipases play a central role in regulating this balance between the storage and degradation/utilization of fatty acids within lipid droplets (48), we investigated this interface in further detail. This resulted in the identification of the lipid droplet-specific lipase ATGL, through phosphorylation-mediated activation, as the primary target of linoleic acid, resulting in the observed accumulation of free fatty acids. Studies designed to delineate the specific upstream signaling pathways contributing towards PUFA-mediated ATGL activation in GBM lipid droplets are ongoing.

We went on to explore the intra-tumoral heterogeneity of this metabolic phenotype in GBM, along with its therapeutic implications. This line of investigation is supported by our previous work, which identified this phenotype as being a requisite adaption that confers GBM cells the ability to acclimate to its dynamic microenvironment(41) and the observation that the accumulation of lipid droplets in GBM organoids are regionally confined to the peritumoral core of an individual tumor (37), which was validated in our mouse model. As we observed not all GBM cells grown in culture harbored the metabolic phenotype of enhanced lipid metabolism, which we defined as an accumulation of lipid droplets, using ow-cytometry, we sorted these cells as lipid droplet ‘high’ and ‘low’. Consistent with the above studies, only lipid droplet ‘high’ cells were responsive to PUFA-induced anti-tumor activity. The biological basis of this intra-tumoral heterogeneity in GBM cells grown in culture is still unclear. One possibility is that despite being a single-cell line, it is composed of a molecularly heterogeneous group of cells, with only a certain population being hardwired with this phenotype. Another possibility is that this phenotype represents some form of transitory state in an individual cell, adapting in some way to its surrounding conditions. In addition to evaluating cells in culture, we grew GBM cells as tumor organoids to provide an additional perspective on the intra-tumoral heterogeneity of this phenotype and its therapeutic implications. Consistent with previous studies(37) and our *in vivo* model, lipid droplets were enriched in the core of GBM organoids. This observed regional heterogeneity supports our overarching hypothesis, being, this phenotype serves as an adaption to its microenvironment. Specifically, the tumor core represents a nutrient-deprived region, where GBM cells likely rely upon the active import of macromolecules from the microenvironment to serve as metabolic substrates. Based on these findings, we hypothesized that cells that have adapted to reside in the tumor core would be sensitive to the antitumor activity of PUFA, which was confirmed in our model. Collectively, these findings provide a framework to design rational combinatorial treatment regimens to target intratumoral heterogeneity in GBM.

We next evaluated combinatorial strategies that may further enhance PUFA-mediated anti-tumor activity in GBM. As our work and others have demonstrated potent enhancement of RT response when combined with the KD, as an initial investigation, we evaluated the interaction between the panel of fatty acids and RT in GBM cell lines in vitro. Consistent with what was observed when tested as a single agent, only the PUFA linoleic acid demonstrated the ability to enhance RT response. We went on to uncover an intriguing mechanism involving the unique chemical structure of PUFAs that likely plays a contributory role in this observed synergy. The carbon-carbon double bonds in PUFAs make them particularly susceptible to free radical-induced lipid peroxidation. As RT generates an abundance of free radicals through ionization, we hypothesized that the combination would lead to further increases in lipid peroxidation, which we went on to demonstrate. This enhancement was rescued by the antioxidant NAC, further supporting the observed synergy between PUFAs and RT is a direct consequence of oxidative stress. As another strategy to enhance PUFA-mediated anti-tumor activity in GBM, we examined combinatorial approaches designed to modulate lipid droplet dynamics. Specifically, as we demonstrated that PUFAs have the unique ability to modulate the lipolysis of lipid droplets, leading to an accumulation of free fatty acids and lipid peroxidation, we hypothesized that impairing the ability of lipid droplets to import these exogenous fatty acids would further enhance the anti-tumor activity of PUFAs in GBM. Consistent with this hypothesis, combining inhibition of DGAT1, which has been shown to attenuate the formation of lipid droplets in GBM cells (35), enhanced the anti-tumor activity of the PUFA linoleic acid, supporting the concept that rational combinations designed to target lipid droplet homeostasis may enhance therapeutic response in GBM.

In addition to a sound understanding of the mechanistic underpinnings contributing towards the antitumor activity of a specific diet in a specific tumor, another factor hindering the successful translation of diet modification into clinical application is long-term adherence to a particular diet. For example, in epilepsy patients, the mean adherence rate of adults to a KD was 63.9% and dropped to 37.7% at 36 months (63). Similar challenges of adherence to a KD were observed when tested in a type 2 diabetes population (64). Further, there is a lack of systematic methods for monitoring adherence to a particular diet, which would be critical for evaluating efficacy of diet modification in the context of clinical trials. One can speculate these challenges would be further intensified in GBM patients, who are often on corticosteroids to manage peritumoral edema, leading to common side effects of increased appetite and elevated blood glucose levels. As our mechanistic-based work identified direct anti-tumor activity of PUFAs in GBM, we hypothesized a PUFA-rich diet could have anti-tumor activity independent of carbohydrate restriction and/or systemic ketosis in mice, which could facilitate adherence. To test this, we developed a PUFA-rich diet formulation. Although the total percentage of kcal from fat in this modified diet was ~ 60%, compared to ~ 90% in the KD, PUFAs made up the majority of these fatty acids and was comparable to the quantity of PUFAs in the KD. This modified diet allowed for a ~ 7-fold increase in carbohydrates, and as expected, did not lead to systemic ketosis. Consistent with our hypothesis, a modified, PUFA-rich diet demonstrated both independent anti-tumor activity and potent ability to enhance RT response GBM mouse models. Although only diet modification was tested here, these findings lay the framework for alternative approaches, included PUFA supplementation using more conventional approaches (e.g. fish or ax seed oil pills) rather than modifying an entire diet, which we anticipate would aid in both adherence and, being more readily quantifiable, translatability for systemic testing in the context of clinical trials.

## METHODS

### Cell lines

MES83 and PN19 cells (kindly provided by Dr. Ichiro Nakano) were cultured in neurosphere complete media consisting of DMEM/F12, GlutaMAX^™^ (Gibco, Grand Island, NY) media supplemented with B-27 (Gibco, Grand Island, NY), human-rFGF (PeproTech, Rocky Hill, NJ), Heparin (STEMCELL Technologies Inc., Cambridge, MA), and human-rEGF (PeproTech, Rocky Hill, NJ). TRP cells were grown in MEM media supplemented with *MEM non-essential amino acids* (Gibco, Grand Island, NY) and 10% FBS (Gibco, Grand Island, NY) (44, 65). U251 cells were purchased from ATCC and were grown in DMEM media (Corning, Manassas, VA) supplemented with 10% FBS. HEK293T cells were grown in DMEM media supplemented with 10% FBS and GlutaMAX^™^ (Gibco, Grand Island, NY). Cell viability was assessed using the trypan blue dye exclusion test. For *in vitro* radiation studies, cells were treated in a Cabinet Irradiator (Xstrahl Inc., GA, USA).

### Reagents

10% bovine-serum albumin (BSA) in DPBS, BSA conjugated oleic and linoleic acid, diethylumbelliferyl phosphate (DEUP), N-acetylcysteine (NAC), and *atglistatin* were purchased from Sigma Aldrich (St. Louis, MO). BSA and BSA-conjugated palmitate were obtained from Cayman Chemicals (Ann Arbor, MI).

### Animal Handling

All *in vivo* experiments were conducted in compliance with institutional guidelines and approved by the Institutional Animal Care and Use Committee of Beaumont Health. Orthotopic xenografts of MES83 and U251 tumors were established in female NU/NU mice (Charles River Laboratories, USA) using previously described methods (46). TRP tumors were orthotopically established in C57BL/6 mice (Jackson Laboratories). Mice were randomly assigned to treatment or control groups using a random number generator. Investigators performing outcome assessments were blinded to group allocation to reduce bias. Mice that exhibited signs of illness, excessive weight loss (>20% of body weight), or abnormal behavior prior to treatment initiation were excluded from the study. Radiation was administered using the Faxitron (Faxitron X-Ray Corp., Wheeling, IL, USA) with custom lead shield designed to shield the remaining body (46). Survival was analyzed using Kaplan-Meier curves. Mice were euthanized upon reaching the endpoint criteria.

### Diet

All animals in all experiments were fed *ad libitum*. Food was monitored and changed daily. The compositions of the diet are as follows:

Standard diet: Control group maintained on a standard mouse chow diet (Teklad 2020x for C57BL/6 and Tekled 2020sx for NU/NU mice).

Ketogenic diet with a 4:1 ratio was purchased from Envigo (WI, USA) or Bioserve (NJ, USA). The sources of fat in the ketogenic diet were lard, anhydrous milk fat, and corn oil. Nutrients (% kcal): Protein (8.4), Carbohydrate (1.4), Fat (90.3). Fat (kcal/gram): PUFA (1.3), MUFA (2.4), Saturated (2.74).

PUFA-rich modified diet was purchased from Envigo (WI, USA). Corn and axseed oil were used as fat sources to prepare the custom-modified diet. Nutrients (% kcal): Protein (29.9), Carbohydrate (10.0), Fat (60.0). Fat (kcal/gram): PUFA (1.92), MUFA (0.705), Saturated (0.341).

### Blood ketone and glucose analysis

Serum ketone and glucose levels were measured using the Precision Xtra (Abbott, CA, USA) blood and ketone monitoring system, following the manufacturer’s instructions.

### Metabolomic proling and data analysis

Metabolites were analyzed using the MxP Q500 kit as directed in the manufacturer’s instructions (biocrates Life Sciences, AG, Innsbruck, Austria). Brie y, tissue was lyophilized, extracted using 100% isopropanol, homogenized, and centrifuged to collect the extracted supernatant. Samples and standards were included in a premix of phenylisothiocyanate for derivatization and subsequently dried under nitrogen. Samples were extracted in 5 mM ammonium acetate in methanol, and the extracts were collected by centrifuging the preparation plate. Sample extracts were diluted with H_2_O (1:1) for the liquid chromatography (LC) phase of the analysis. For the ow injection analysis (FIA), the sample extract was mixed with the kit solvent in a separate plate, and the QC sample extract was mixed with the kit solvent.Sample extracts were analyzed using a Waters I-class UPLC unit coupled with a Waters Xevo-TQ-S (Waters Corporation, Milford, MA, USA). For UPLC analysis, sample extracts were separated using the MxP Quant 500 C18 column with an attached guard and precolumn mixer (biocrates Life Sciences, AG, Innsbruck, Austria). The mobile phase consisted of A: H_2_0 and formic acid (0.2%); B: MeCN and 4-formic acid (0.2%) delivered at a ow rate of 0.8mL/min with a gradient of B: 0–100% over 4.50 minutes. Eluent %B was increased to 1.00mL/min ow rate and maintained at 100% for 30 seconds, followed by a rapid return to the initial conditions for 70 seconds to equilibrate the column. Both positive and negative mode gradients were 5.80 minutes long. The negative mode acquisition gradient differed from the positive mode with a difference in %B composition between 2.00 and 4.50 minutes. The injection volume was 5 mL for positive data acquisition and 15 mL for the negative run. The wash solvent composition consisted of H2O: MeOH: MeCN: IPA (v/v). Q500 Kit offers direct ow injections (FIA) for lipid analysis. An isocratic method was performed using the kit-provided solvent (290 mL MeOH: 1 ampule of FIA additives). The isocratic mobile phase (B: 100% MeOH) was delivered at a low ow rate of 0.03 mL/min. The injection volume was 20 mL for both positive and negative mode acquisitions. All data were extracted using the MetIDQ software. Metabolites below the limit of detection (LOD) in all treatment conditions or were identified in less than 50% of samples in a group were not reported. The Differential Abundance (DA) score was calculated as described previously (40). Heatmap and PLS-DA were generated using MetaboAnalyst (66).

### Fatty acid uptake

Evaluation of fatty acid uptake was performed as previously described (40). Brie y, cells were cultured in a 96-well plate overnight, and media was replaced with HBSS media. The cells were then incubated for 1 h in a humidified 37°C incubator with 5% CO_2_. A mixture of fatty acid-BSA BODIPY (5 mM HEPES, 10 mM BODIPY, 5 mM fat-free BSA, and 4 mM trypan blue) was added to the cells, and fluorescence was measured at excitation and emission wavelengths of 485 and 528, respectively using *a SpectraMax Gemini* EM Microplate Reader (Molecular Devices, CA, USA).

### Confocal microscopy analysis of lipid droplets

Cells were stained with BODIPY 493/503 (0.5 μM) for 15 min followed by counterstain with DAPI (BioRad, Hercules, CA) and visualized by confocal microscopy (Nikon Eclipse Ti, 40 or 60x oil). Images were processed using Fiji software (NIH), as previously described (35, 67).

### Free fatty acid analysis

Intracellular free fatty acids were analyzed using the Free Fatty Acid Quantitation Kit (Sigma Aldrich, MA, USA) per the manufacturer’s instructions. Brie y, cells were homogenized in a 1% (w/v) Triton X-100 (EMD chemicals, NJ, USA) in chloroform solution (Sigma Aldrich, MA, USA), followed by centrifugation at 13,000 × g for 10 minutes to remove insoluble material. The organic phase (lower phase) was collected and air-dried at 50°C to remove chloroform, followed by vacuum drying for 30 minutes to remove any trace chloroform. The dried lipids were then dissolved in fatty acid assay buffer by vortexing extensively for 5 minutes. Free fatty acids were quanti ed using colorimetric assay methods using a *XMARK*microplate spectrophotometer (Bio-rad, Hercules, CA).

### Flow cytometric analysis of lipid droplets and apoptosis

Cells were stained with BODIPY 493/503 (1 μM) to evaluate lipid droplets (68). The samples were then analyzed using a BD FACS Canto II ow cytometer (Becton Dickinson; Mountain View, CA), and the analysis was carried out using FlowJo v10 Software (FlowJo, LLC; Ashland, OR). Apoptotic cells were assessed through staining with Annexin V (Invitrogen, Carlsbad, CA), and 7AAD (Calbiochem, San Diego, CA) as per the manufacturer’s protocol.

### Western blot

Western blot was performed using methods previously described (8). A custom antibody against pATGL was generated by ABclonal (Woburn, MA). Antibody against ATGL was purchased from Cell Signaling Technology (Danvers, MA). Antibody against phosphorylated ATGL was obtained from Abcam (ab135093, Cambridge, MA). Antibody against TFRC was obtained from Abcam (ab214039, Cambridge, MA). HRP-conjugated secondary antibodies were obtained from Sigma Aldrich (St. Louis, MO).

### Lipid peroxidation

Lipid peroxidation was determined by levels of malondialdehyde (MDA) using an MDA assay kit (ab118970, Abcam). Brie y, cell supernatants after homogenization were mixed with thiobarbituric acid (TBA) and heated for 1 h at 95°C. After cooling on ice, the absorbance of the samples was measured at 532 nm. The content of MDA was calculated by a standard concentration curve.

### Ferroptosis

Ferroptosis was determined by iron content using an iron assay kit (ab83366, Abcam). Brie y, cells were collected, homogenized (in cold iron assay buffer), and centrifuged. 5 μL of iron reducer was added to samples and were incubated for 30 min at 37 °C. Next, 100 μL of iron probe was added and samples were incubated for 60 min at 37 °C in the dark. The absorbance at 593 nm was measured using a microplate reader.

### Tumor organoids

Tumor organoids were cultured using methods previously described (37). Brie y, GBM organoids were created by suspending 10,000 tumor cells in Matrigel^®^ (Corning, NY) and forming 20 μL pearls on an Organoid Embedding Sheet (Stemcell Technologies, Cambridge, MA), before culturing them in 6-well or 10 cm plates with shaking in neurosphere complete media.

### Tissue processing

Mouse brains and organoids were immersed in 4% (wt/vol) paraformaldehyde (PFA) overnight for fixation, cryoprotected in 30% sucrose, embedded in OCT, and sectioned at a thickness of 10 μm using a cryostat.

### Oil Red O staining

Lipid droplets in tumors and tumor organoids were measured by Oil Red O staining. Frozen sections were air-dried at room temperature, washed with PBS, rinsed with 60% isopropanol, and stained with Oil red O working solution (freshly prepared from 60 ml of Oil Red O solution from Sigma and 40 ml distilled water) for 15 minutes. After staining, slides were rinsed with 60% isopropanol, followed by 2 rinses of distilled water, counterstained with Modi ed Mayer’s Hematoxylin (Abcam, Cambridge, MA), rinsed in tap water, and mounted in aqueous mounting medium (Newcomer Supply, Middleton, WI). Sections were visualized under Leica Aperio.

### Immunohistochemistry

Frozen sections were air-dried at room temperature, washed in PBS, and then antigen retrieved in 0.1 M citrate buffer (pH 6, Vector Laboratories, Newark, CA) for 10 min using a steamer. Sections were incubated with 0.3% H_2_O_2_ in methanol for 30 min to block endogenous peroxidase activity. After blocking with PBS containing 5% normal goat serum (Vector Laboratories, Newark, CA) and 0.3% Triton X-100 (Calbiochem, San Diego, CA) for 1 hour, sections were incubated with primary antibody (cleaved caspase 3, Cell signaling technology, Danvers, MA) diluted in Antibody Diluent Reagent Solution(Life Technologies, Carlsbad, CA) overnight at 4 °C. Sections were washed with PBS, incubated with SignalStain^®^ Boost IHC Detection Reagent (Cell signaling technology, Danvers, MA) for 1 hour at RT. DAB (Vector Laboratories, Newark, CA) was used as a chromogen and sections were counterstained using methyl green (Vector Laboratories, Newark, CA). Sections were visualized under Olympus APX100 system.

### Cell sorting

For ow-sorting, we followed methods described by Herms et al., 2013 (69). Brie y, cells were collected and washed with PBS. The cell density was adjusted 1×10^7^cells/ml, filtered with a cell-strainer of 70 μm, and sorted by side scatter (SSC-A) values using a FACS Aria Fusion (BD Bioscience, NJ, USA), collecting the population in the extremes (high SSC and low SSC). After sorting, cells were stained with BODIPY to validate lipid droplet.

### shRNA transfection

Inducible scrambled, and DGAT1 shRNA plasmids were obtained from Biosettia (San Diego, CA) and transformed into TOP 10 E. coli competent cells (Tiangen, Beijing, China). The positive clone was selected and subsequently grown in LB broth overnight. The PureYield Plasmid Maxiprep system (Promega, WI, USA) was used to extract the plasmid. HEK293T cells were transfected with DGAT1 or scrambled plasmid along with psPAX2 (Addgene, MA, USA) and pMD2.G (Addgene, MA, USA) using Lipofectamine 2000 (Invitrogen, MA, USA). Lentivirus was collected after 48, 72, and 96 hours. Stable cell lines were then transfected with the lentivirus, followed by blasticidine selection. Doxycycline was used to induce knockdown, which was con rmed by qRT-PCR.

### Real-time PCR

Expression of genes was determined using SsoAdvanced Universal *SYBR Green* Supermix (Bio-rad, Hercules, CA) and ViiA 7 Real-Time PCR System (Applied Biosystems, Foster City, CA). RNA was isolated using an Aurum^™^ Total RNA Mini Kit (BioRad, Hercules, CA), followed by cDNA preparation from total RNA using an iScript cDNA Synthesis Kit (BioRad, Hercules, CA). Primer pairs used in quantitative real-time PCR experiments: DGAT1 Forward 5’-TCGATGATGCGTGAGTAGTCC-3’ Reverse 5’-CAATCTGACCTACCGCGATCT-3’, β-Actin Forward 5’-GGATCAGCAAGCAGGAGTATG-3’ Reverse 5’AGAAAGGGTGTAACGCAACTAA-3’,

### Statistical analysis

Sample sizes were determined using power analysis based on prior survival studies. A minimum of n=6 per group was chosen to detect a 20% difference in survival with 80% power at α=0.05. A log-rank test was used for survival analyses, assuming proportional hazards and independent survival times. Statistical data analysis (one-way ANOVA with Tukey’s test for multiple comparisons, or T-test for pairwise comparisons) was performed using Origin Pro software (Origin Lab Corporation).

## Supplementary Material

This is a list of supplementary les associated with this preprint. Click to download.

- UneditedBlotsandMicroscopy.pptx
- SupportingDataValues.xlsx
- SupplementaryTable1.pdf
- FigS1.tif
- FigS2.tif

## Data Availability

A Supporting Data Values le containing all the underlying values for data presented in the manuscript.
